# Metropolitan Hospital Sunday

**Published:** 1903-08-08

**Authors:** 


### METROPOLITAN HOSPITAL SUNDAY.

### NO HOSPITAL APPEALS IN HOSPITAL SUNDAY WEEK.

A meeting of the Council was held at the Mansion House on July 30th to receive the report of the committee of distribution, to order payment of awards to hospitals and dispensaries, and to consider a recommendation of the General Purposes Committee that an addition should be made to Law IX. of the laws of the constitution.

The report showed that the committee of distribution recommended awards this year to 209 institutions. The total amount of collections up to the present time was £60,900. The distribution of £58,386 11s. 8d. to 154 hospitals and to 56 dispensaries was recommended; 211 institutions, or seven more than last year, had applied for grants. The committee were unable to recommend a grant this year to one hospital, and one hospital was considered ineligible. The outlay on repairs and improvement of buildings had not been included in arriving at the financial basis of each institution, so that the annual distribution was wholly awarded for the maintenance of patients.

The special attention of the Committee of Distribution had been directed to the constantly increasing expenditure of hospitals in the maintenance of patients. The average cost per week of each patient treated at 21 of the principal hospitals amounted to £1 16s. 6½d. in 1902. In 1897 the average was £1 13s. 1½d. In 1892 the average was £1 12s. 3d. The difference in the 10 years of 4s. 3½d. represented in these 21 hospitals alone an increase of over £44,000 for a single year. While recognising the fact that the expense must necessarily have increased to some extent with the progress of scientific treatment and nursing, the committee still thought that the expense in some hospitals was higher than justified by these considerations. The committee reduced the bases of award in 38 cases. The collections at St. Paul's on the occasion of their Majesties' visit amounted to £5,500. In spite of the very inclement weather on Hospital Sunday the amount nearly approached the collection of 1902, the record year.

The chair was taken by Sir Sydney Waterlow, who said that their principal business was to submit the thirty-first report of the Distribution Committee, and he recalled the foundation of the fund in 1872, since which time the sum of £1,239,933 had been collected and distributed under its auspices. He thought he was justified in stating that the Hospital Sunday Fund had been a success. The collections this year were very satisfactory, more so than in any previous year. More money had been collected in the churches, and there were still further sums to come in before the end of their financial year in October. The collections this year had been materially increased by the visit paid by the King and Queen to St. Paul's, when £5,500 was collected, and the fact that his Majesty had desired this sum to be paid in to the treasurer of this Fund was the best evidence they could have that the King had confidence in the way in which the money was divided amongst the hospitals, dispensaries, and other institutions. There was a larger number of congregations contributing this year, which was also very satisfactory, and while they had more than 2,000 places of worship supporting this fund they might feel that it rested on a solid basis. The committee had had a very busy time this year and, in addition to other duties had received eleven deputations from various hospitals. The committee had for a long time been much exercised concerning the great cost per bed for in-patients, but it was only in their power to report the increase in cost and let the public consider whether an increase of 3s. 5d. per bed in five years and of 4s. 3½d. in ten years was too much. One point the committee had carefully considered was the difficulty of determining the cost per out-patient and they had determined to call a conference as early as was possible of experts and those who had the greatest knowledge and experience of the workings of hospitals, for the purpose of arriving at some conclusion as to the best method of determining the cost of out-patients, which materially affected the cost of in-patients. The collections had also been very largely increased by the money received from Mr. George Herring, to whom they could not be too grateful. Last year he had given £11,575, and this year £11,600.

Sir Sydney Waterlow then moved that the report be approved and the awards recommended be paid as soon as possible.

This resolution was seconded by the Rev. Prebendary Ridgeway, who, alluding to the inclement weather on Hospital Sunday, said that one moral was clearly proved, and that was that those churches where the practice of bringing the fund before their congregations prior to the actual Sunday had been adopted had suffered the least.

Sir Henry Burdett, who was the next speaker, said that this being the first occasion on which the distribution committee had dealt with expenditure, he would like to draw attention to some figures in the report as to which he would ask for a little explanation. He noticed, to begin with, that they had distributed this year a sum of £600 more than last year; the actual figures were £57,800 in 1902 and £58,386 in 1903; but he had spent such time as he could spare in trying to find out where the extra money was given to, and had not succeeded. It had not been given to the dispensaries, and he always felt that the fund might give a little more to the dispensaries, seeing that they were the oldest unit of out-patient work in London and the one branch of the medical system that had gone on from the first conducting their business on business principles. It was a most economically conducted system, and also, what was really more important in this metropolis, the one system in which they were assured by the safeguards enforced that not one penny went to the relief of the unworthy. The dispensaries also visited patients in their own homes, and by reporting insanitary dwellings to the authorities were of the greatest benefit from a hygienic point of view. He observed that they based their awards this year on the cost per bed. He found that at the hospital with the greatest expenditure, St. George's, the cost per bed was £2 14s. 10d. per week; their award had been reduced by £780. The next largest expenditure, at the Brompton Hospital, was £2 4s. 3d. per patient, and their deduction was £90. The cost at King's was £2 1s. 9d. per patient, and the committee had added £120 to their grant, and in the case of the Middlesex Hospital, with a cost of £2 1s. 1½d., they had added £100 to the actual grant, and in addition, for the first time this year, had given an extra £325 under special hospitals towards the cancer department, so that the Middlesex, with a cost of £2 1s. 1½d. had an extra grant of £425. At Guy's and at the London Hospital the cost was respectively £2 0s. 5d. and £2 per bed, and from Guy's grant was deducted £600, while £650 was deducted from the London Hospital grant. These diminished grants of course should be added to the £600 extra money that they had to give away, and what he wanted to know was how this extra money had been given.

The Chairman replied that no one appreciated more than he did the splendid work done by the dispensaries, and personally would have been glad to be able to deal a little more generously with them, but the committee could not resist the feeling that the public subscribed their money with the idea that it went to the hospitals and not to the dispensaries. With regard to the extra money, the fact must not be lost sight of that in distributing the money they did so according to the cost per bed and also according to the needs and merits of an institution.

Sir Edmund Hay Currie, the secretary, said that the money had gone to those hospitals which were more economically conducted. Those which were expensively conducted had their bases reduced and those economically carried on had their bases increased. Thirty-eight hospitals had been reduced.

The Ven. Archdeacon Sinclair then moved an addition to Law IX. which had been recommended by the General Purposes Committee and ran as follows:—"In the further event of a particular hospital, dispensary or institution fixing any day or days in the week immediately preceding Hospital Sunday in any year for the purpose of pressing the claims of such institution upon the public, it shall be an instruction to the Committee of Distribution when determining the award to such institution to take into consideration the amount received from such an appeal and the loss, if any, resulting therefrom to this Fund." The Archdeacon said that this alteration was not vindictive, but was made in order to guarantee that the week preceding Hospital Sunday should be kept free from appeals such as had been made by the London Hospital this year. The press, in consequence of their columns having been so much occupied with the London Hospital appeal, had been unable to devote so much space to this Fund as they had heretofore done.

The motion was seconded by Mr. F. H. Norman, and Sir Stephen Mackenzie rose to point out that the London Hospital, of which he was a governor, was not a wealthy institution, as the Archdeacon had stated, and also that the date of appeals was usually fixed some months ahead.

The Chairman said that the date of Hospital Sunday was known many months previously—*i.e.*, in December in each year.

Sir Henry Burdett remarked that any hospital that issued appeals in the days that courtesy and custom had assigned to the Hospital Sunday Fund must do itself immeasurable injury.

Mr. C. E. Tritton, M.P., moved that the cordial thanks of the Council should be given to Mr. George Herring for his generous and continued interest in the Fund, his munificent offer having to a large extent succeeded this year in raising the collections in places of worship to a record amount. This was seconded by the Rev. E. H. Pearce.

A vote of thanks to Sir Sydney Waterlow and the members of the Committee of Distribution was moved by Mr. Thos. Bryant, F.R.C.S., and seconded by Rev. W. H. Harwood.

Mr. Christie then moved a vote of thanks to the Press for advocating the cause of the Fund, which was seconded by Sir Stephen Mackenzie, M.D.

The proceedings terminated with a vote of thanks to the Lord Mayor, proposed by Mr. Alfred Willett, F.R.C.S, and seconded by Sir Sydney Waterlow.

